# Altered proliferative ability of neuronal progenitors in PlexinA1 mutant mice

**DOI:** 10.1002/cne.23806

**Published:** 2015-07-01

**Authors:** William D. Andrews, Kathryn Davidson, Nobuaki Tamamaki, Christiana Ruhrberg, John G. Parnavelas

**Affiliations:** ^1^ Department of Cell and Developmental BiologyUniversity College LondonLondonWC1E 6BTUnited Kingdom; ^2^ Division of Visual Science and Molecular GeneticsInstitute of Ophthalmology, University College LondonLondonWC1E 6BTUnited Kingdom; ^3^ Department of Morphological Neural ScienceGraduate School of Medical Sciences, Kumamoto UniversityKumamoto860 0862Japan

**Keywords:** neuronal migration, Plexin, interneurons, proliferation, forebrain

## Abstract

Cortical interneurons are generated predominantly in the medial ganglionic eminence (MGE) and migrate through the ventral and dorsal telencephalon before taking their final positions within the developing cortical plate. Previously we demonstrated that interneurons from Robo1 knockout (*Robo1^−/−^*) mice contain reduced levels of neuropilin 1 (Nrp1) and PlexinA1 receptors, rendering them less responsive to the chemorepulsive actions of semaphorin ligands expressed in the striatum and affecting their course of migration (Hernandez‐Miranda et al. [2011] J. Neurosci. 31:6174–6187). Earlier studies have highlighted the importance of Nrp1 and Nrp2 in interneuron migration, and here we assess the role of PlexinA1 in this process. We observed significantly fewer cells expressing the interneuron markers *Gad67* and *Lhx6* in the cortex of *PlexinA1* ^−/−^ mice compared with wild‐type littermates at E14.5 and E18.5. Although the level of apoptosis was similar in the mutant and control forebrain, proliferation was significantly reduced in the former. Furthermore, progenitor cells in the MGE of *PlexinA1* ^−/−^ mice appeared to be poorly anchored to the ventricular surface and showed reduced adhesive properties, which may account for the observed reduction in proliferation. Together our data uncover a novel role for PlexinA1 in forebrain development. J. Comp. Neurol. 524:518–534, 2016. © 2015 The Authors The Journal of Comparative Neurology Published by Wiley Periodicals, Inc.

The medial ganglionic eminence (MGE) is the major source of interneurons to the mammalian cerebral cortex (Lavdas et al., 1999; Wichterle et al., 2001; Hansen et al., 2013). En route to the cortex, migrating MGE‐derived interneurons encounter the developing striatum and avoid it (Marin and Rubenstein, 2003; Métin et al., 2006). Work over the last decade has identified some of the cellular and molecular mechanisms that guide their migration through the ventral telencephalon (for review see Hernandez‐Miranda et al., 2010; Faux et al., 2012). These include class 3 semaphorin ligands and their receptors, the neuropilins (Nrps) and plexins.

Earlier studies have shown that migrating cortical interneurons express Nrp receptors (Marin et al., 2001; Tamamaki et al., 2003a). Blocking Nrp1 function with either anti‐Nrp1 antibody (Tamamaki et al., 2003a) or Nrp1 dominant negative focal electroporation in slice cultures disrupted their migration, with a significant number straying into the striatum (Marin et al., 2001). Similarly, loss of Nrp2 function in *Nrp2^−/−^* transgenic mice resulted in more cortical and striatal (neuropeptide Y [NPY]‐expressing) interneurons in the striatum (Marin et al., 2001). More recently, we have found that interneurons in mice lacking the roundabout receptor *Robo1* have reduced levels of Nrp1 and PlexinA1 compared with control littermates and are less responsive to Sema3A and Sema3F, resulting in their aberrant migration through the striatum (Hernandez‐Miranda et al., 2011). This raises the question of whether the Nrp coreceptor PlexinA1 is important for cortical interneuron migration.

Here, we quantified the number and distribution of interneurons in the cortex of *PlexinA1^−/−^* mice and littermate controls in the middle and late stages of corticogenesis. We found significantly fewer cells in mice lacking the receptor, suggesting disrupted migration and/or reduced generation in the MGE. Further experiments showed a marked decrease in proliferation in ventral and dorsal forebrain, suggesting a reduction in the number of interneuron and pyramidal cell progenitors. Nestin staining in the proliferative zones of the MGE confirmed not only the reduction of progenitor cells in the knockout but also altered morphology, with cells often lacking attachments to the ventricular surface. Furthermore, adhesion assay experiments showed reduced attachment in *PlexinA1^−/−^* mice compared with controls. Together our data suggest that reduced adhesiveness of interneuron progenitors in *PlexinA1^−/−^* mice may underlie the observed reduction in proliferation, resulting in fewer interneurons (and pyramidal cells) in the cortex during development.

## MATERIALS AND METHODS

### Animals

All experimental procedures were performed in accordance with the U.K. Animals (Scientific Procedures) Act 1986 and institutional guidelines. Wild‐type animals were C57/bl6J mice obtained from Charles River, Ltd. *PlexinA1^−/−^* and *GAD67‐GFP^neo‐^* mice were generated as described previously (Yoshida et al., 2006 [PMID: 17145500]; Tamamaki et al., 2003b [PMID: 14574680]). PlexinA1 mice were genotyped by polymerase chain reaction (PCR) with the following primers: WT‐forward (5′‐CCTGCAGATTGATGACGACTTCTGC‐3′), WT‐reverse (5′‐TCATGCAGACCCAGTCTCCCTGTCA‐3′), product size 200 bp; and mutant‐forward (5′‐GCATGCCTGTGACACTTGGCTCACT‐3′), mutant‐reverse (5′‐CCATTGCTCAGCGGTGCTGTCCATC‐3′), product size 600 bp. The day on which the vaginal plug was found was considered embryonic day (E) 0.5. Animals of both sexes were used in our experiments.

### In situ hybridization

For in situ hybridization and immunohistochemistry, embryonic brains were dissected in phosphate‐buffered saline (PBS) and fixed in 4% paraformaldehyde (PFA), made by dissolving PFA in PBS for 4–8 hours at room temperature (RT). After fixation, embryonic brains were cryoprotected in 30% sucrose in diethyl pyrocarbonate (DEPC)‐treated PBS, embedded and frozen in a mixture of 15% sucrose/50% Tissue‐Tek OCT (Sakura Finetek), and sectioned in the coronal plane at 20 µm with a cryostat (Bright Instruments). Sections were dried at RT for 2 hours before overnight incubation at 65°C in hybridization buffer (a DEPC‐treated solution containing 200 mM NaCl, 5 mM EDTA, 10 mM Tris, pH 7.5, 5 mM NaH_2_PO_4_ · 2H_2_O, 5 mM Na_2_HPO_4_ [Sigma‐Aldrich, St. Louis, MO]; 50% deionized formamide [Ambion, Austin, TX]; 0.1 mg/ml RNase‐free yeast tRNA [Invitrogen, Carlsbad, CA]; 1× RNase/DNase‐free Denhardt's [Invitrogen]; 10% dextran‐sulfate [Sigma‐Aldrich]) containing 100–500 ng/ml DIG‐labeled RNA probes. Antisense probes were generated as described in Table 1. After hybridization, sections were washed three times in 50% formamide 1× SSC (Ambion) and 0.1% Tween‐20 (Sigma‐Aldrich) at 65°C and twice at RT in 1× MABT (20 mM maleic acid, 30 mM NaCl, 0.1% Tween‐20 [Sigma‐Aldrich]) before incubating in blocking solution [MABT containing 2% blocking reagent [Roche. Indianapolis, IN] and 10% normal goat serum [Vector, Burlingame, CA]), followed by overnight incubation in alkaline phosphatase‐conjugated anti‐DIG antibody (1:1,500; Roche). Nitroblue tetrazolium chloride/5‐bromo‐4‐chloro‐3‐indolyl phosphate (Roche) diluted 1:1,000 in MABT containing 5% polyvinyl alcohol (VWR International) was used for the colorimetric detection and Fast Red (Roche) dissolved in 100 mM Tris (pH 8.0) and 400 mM NaCl for fluorescent color detection by incubation at 37°C. Fluorescence in situ hybridization was followed by immunohistochemical detection of green fluorescent protein (GFP) as described below. Sections were mounted with Glycergel mounting medium (Dako, Carpinteria, CA).

**Table 1:** In Situ Hybridization Probes

Antisense probe	Source	Restriction enzyme	RNA polymerase
*PlexinA1*	Dr. Nina Perälä, University of Helsinki (Finland)	XhoI	T3
*Lhx6*	Dr. Nicoletta Kessaris, UCL, UK	Not1	T3
*ER81*	Dr. Thomas Jessell, Columbia University (U.S.)	SpeI	T7
*GAD67*	Dr. Brian Condie, University of Georgia (U.S.)	XhoI	T3

### Immunohistochemistry

Embryonic brain sections were washed in PBS, blocked in a solution of 5% normal goat serum (v/v; Sigma‐Aldrich) containing 0.1% Triton X‐100 (v/v; Sigma‐Aldrich) in PBS at RT for 2 hours. They were subsequently incubated in primary antibodies at RT for 2 hours and, then, at 4°C overnight. After incubation in primary antibodies, sections were washed in PBS, incubated in biotinylated anti‐species (1:250; Vector) for 2 hours, and processed via immunohistochemistry protocols described previously (Andrews et al., 2008).

### Antibody characterization

Details of the antibodies used in this study are summarized in Table 2.

**Table 2:** Antibodies Used^a^

Antibody ID	Antibody name	Antibody target	Vendor	Catalog No.	Clonality	Clone ID	Host organism	Proper citation	Reference
AB_306886	Mouse anti‐BrdU monoclonal antibody, unconjugated, clone IIB5	BrdU	Abcam	ab8955	Monoclonal antibody	Clone IIB5	Mouse	(Abcam catalog No. ab8955, RRID: AB_306886)	Morris and Solomon, 2004
AB_10000340	Rabbit anticalbindin D‐28k antibody	Rabbit anticalbindin D‐28k	Swant	CB 38	Polyclonal antibody		Rabbit	(Swant catalog No. CB 38, RRID: AB_10000340)	Suárez et al., 2006
AB_331453	Cleaved caspase‐3 (Asp175) (5A1E) rabbit mAb	Cleaved caspase‐3 (Asp175) (5A1E) rabbit mAb	Cell Signaling Technology	9664S	Polyclonal antibody		Rabbit	(Cell Signaling Technology catalog No. 9664S, RRID: AB_331453)	Tokami et al., 2013
AB_2261231	CDP (M‐222) antibody	CDP (M‐222)	Santa Cruz Biotechnology	sc‐13024	Polyclonal antibody		Rabbit	(Santa Cruz Biotechnology catalog No. sc‐13024, RRID: AB_2261231)	Arellano et al., 2012
AB_2064130	Rat anti‐Ctip2 monoclonal antibody, unconjugated, clone 25B6	Ctip2	Abcam	ab18465	Monoclonal antibody	Clone 25B6	Rat	(Abcam catalog No. ab18465, RRID: AB_2064130)	Stillman et al., 2009
AB_2107107	Rabbit anti‐FOXP2 polyclonal antibody, unconjugated	FOXP2	Abcam	ab16046	Polyclonal antibody		Rabbit	(Abcam catalog No. ab16046, RRID: AB_2107107)	Campbell et al., 2009
AB_10000240	Chicken anti‐GFP antibodies	GFP	Aves Labs	GFP‐1020	Polyclonal antibody		Chicken	(Aves Labs catalog No. GFP‐1020, RRID: AB_10000240)	Xu et al., 2006
AB_2235915	Mouse anti‐rat nestin monoclonal antibody, unconjugated	rat nestin	Developmental Studies Hybridoma Bank	Rat‐401	Monoclonal antibody		Mouse	(Developmental Studies Hybridoma Bank catalog No. Rat‐401, RRID: AB_2235915)	Wang et al., 2006
AB_1587367	Rabbit anti‐PAX6, unconjugated antibody	PAX6	Millipore	AB2237	Polyclonal antibody		Rabbit	(Millipore catalog No. AB2237, RRID: AB_1587367)	Piper et al., 2011
AB_310177	Rabbit antihistone H3 (phosphorylated at Ser10), mitosis marker polyclonal antibody, unconjugated	Histone H3 (phosphorylated at Ser10)	Millipore	06–570	Polyclonal antibody		Rabbit	(Millipore catalog No. 06–570, RRID: AB_310177)	Wang et al., 2006
AB_2166258	PlexinA1 (H‐60) antibody	PlexinA1 (H‐60)	Santa Cruz Biotechnology	sc‐25639	Polyclonal antibody		Rabbit	(Santa Cruz Biotechnology catalog No. sc‐25639, RRID: AB_2166258)	Cariboni et al., 2007

a BrdU, bromodeoxyuridine; CB, calbindin; CDP, CCAAT displacement protein ovalbumin upstream promoter transcription factor‐interacting protein‐2; FOXP2, Forkhead box protein P2; Pax6, paired box; Ctip2, B‐cell leukemia/lymphoma 11B.

#### Bromodeoxyuridine antibody

A mouse monoclonal antibody raised against bromodeoxyuridine (BrdU) and conjugated to bovine serum albumin (BSA; Abcam, Cambridge, MA; catalog No. ab8955, RRID: AB_306886) was used to immunolabel proliferating progenitor cells in the developing forebrain following injection of BrdU into pregnant dams (Cavanagh et al., 1997). BrdU immunohistochemistry of wild‐type mouse forebrain sections showed no staining.

#### Calbindin antibody

The calbindin D‐28 (CB) antiserum (Swant, Belinzona, Switzerland; catalog No. CB 38, RRID: AB_10000340) recognized a single band of 28 kDa on Western blots of rat brain (manufacturer's data sheet) and stained a pattern of cellular morphology and distribution in the mouse developing cerebral cortex that is identical to that in previous reports (Andrews et al., 2008; Hernandez‐Miranda et al., 2011).

#### Cleaved caspase‐3 antibody

We used a rabbit monoclonal antibody (Cell Signaling Technology, Danvers, MA; catalog No. 9664S, RRID: AB_331453) raised against a synthetic peptide corresponding to amino‐terminal residues adjacent to Asp175 of human caspase‐3 to detect endogenous levels of the large fragment (17/19 kDa) of activated caspase‐3 (Yeh et al., 2014).

#### CDP antibody

CDP (M‐222) is a rabbit polyclonal antibody (Santa Cruz Biotechnology, Santa Cruz, CA; catalog No. sc‐13024, RRID: AB_2261231) raised against amino acids 1111–1332 of CDP/Cux1 of mouse origin. CDP immunohistochemistry of wild‐type embryonic mouse forebrain sections specifically labels upper layer pyramidal neurons (Arlotta et al., 2005; Yeh et al., 2014).

#### Ctip2 antibody

Rat monoclonal antibody (25B6; Abcam; catalog No. ab18465, RRID: AB_2064130) to Ctip2 recognizes an epitope between amino acids 1 and 150 of human CTIP2. Ctip2 immunohistochemistry carried out on wild‐type embryonic mouse forebrain sections specifically labels lower layer pyramidal neurons (Cubelos et al., 2008; Yeh et al., 2014).

#### FOXP2 antibody

Rabbit polyclonal FOXP2 antibody (Abcam; catalog No. ab16046, RRID: AB_210710) raised against a synthetic peptide in the C‐terminus of human FOXP2 was previously shown to immunolabel striatal projection neurons in the developing mouse forebrain (Takahashi et al., 2003; Hernandez‐Miranda et al., 2011).

#### GFP antibody

We used a GFP antiserum (Aves Labs; catalog No. GFP‐1020, RRID: AB_10000240) that recognizes the expected (27 kDa) band on Western blots of GFP‐positive transgenic mouse brain (manufacturer's data sheet). GFP immunohistochemistry, carried out on wild‐type mouse forebrain sections, showed no staining except for weak autofluorescence in the choroid plexus (data not shown).

#### Nestin antibody

Mouse nestin monoclonal antibody (Developmental Studies Hybridoma Bank; catalog No. Rat‐401, RRID: AB_2235915) raised against E15 Sprague‐Dawley rat spinal cord labeled radial glia progenitors, as previously described (Cavanagh et al., 1997).

#### Pax6 antibody

Pax6 rabbit polyclonal antibodies (Millipore, Bedford, MA; catalog No. AB2237, RRID: AB_1587367) raised against the C‐terminus of human PAX6 produced the characteristic labeling of cortical neuronal progenitors on embryonic mouse forebrain tissue, as previously described (Quinn et al., 2007).

#### Phospho‐histone H3 antibody

Phospho‐histone H3 (PH‐3) rabbit polyclonal antibodies (Millipore; catalog No. 06–570, RRID: AB_310177) raised against a linear peptide corresponding to human histone H3 phosphorylated at Ser10 was used to quantify cell proliferation in the developing cortex, as previously described (Andrews et al., 2008; Yeh et al., 2014).

#### PlexinA1 antibody

A PlexinA1 (H‐60) rabbit polyclonal antibody (Santa Cruz Biotechnology; catalog No. sc‐25639, RRID: AB_2166258) raised against amino acids 961–1020 of human PlexinA1, shown to recognize PlexinA1 in brain lysates by Western blotting, stained many cell types on wild‐type, but not knockout, forebrain sections (data not shown).

### Interneuron counts in the cortex

Interneurons labeled with *Lhx6*, *GAD67*, or calbindin were counted in images collected with a Leica DM5000B light microscope. The images were of coronal strips (200 μm wide) spanning the thickness of the neocortex throughout its rostrocaudal extent at different ages (minimum of six sections from each of three animals for each genotype). Each coronal strip was divided into bins arranged parallel to the pial surface that corresponded to the different layers of the developing cortex.

### Proliferation

The mothers of E14.5 embryos were injected intraperitoneally with 50 μg BrdU (Sigma‐Aldrich) per gram of body weight and culled 1 hour later. Brains were then fixed with PFA, and 20‐μm‐thick sections were cut with a cryostat. For BrdU immunolabeling, sections were first incubated with 2 N HCl at 37°C for 30 minutes to unmask the antigen, followed by three washes in PBS. PH‐3 staining was performed as described above. We counted all PH‐3^+^ cells in a 200‐µm‐wide strip perpendicular to the ventricular wall of the VZ and in a 4 × 10^4^‐µm^2^ area within the SVZ of the MGE.

### Dissociated MGE cell cultures

Dissociated cell cultures were prepared from E12.5 mice as described previously (Cavanagh et al., 1997). Briefly, MGEs were dissected out in cold artificial cerebrospinal fluid (ACSF) under a stereomicroscope. They were incubated in neurobasal medium (Life Technologies, Grand Island, NY) containing 0.05% trypsin (Sigma‐Aldrich) and 100 µg/ml DNaseI (Roche) at 37°C for 15 minutes. Trypsinization was quenched with neurobasal medium containing 10% FBS (Life Technologies) at 37°C for 5 minutes. MGEs were then triturated by pipetting until no cellular aggregates were visible. The homogeneous cell suspensions were subsequently pelleted by centrifugation at 1,000*g* for 3 minutes. Cells were resuspended in DMEM/F12 culture media containing B27 supplement, 100 µg/ml penicillin/streptomycin, and 2 mM L‐glutamine (Life Technologies), and 100,000 cells were seeded onto 13‐mm coverslips coated previously with 10 µg/ml poly‐L‐lysine and 10 µg/ml laminin (Sigma‐Aldrich) and incubated in a the humidified incubator at 37°C. On the next day, the culture medium was changed.

### Chemotaxis assays

Chemotaxis assays were performed with a 48‐well Boyden's chamber (NeuroProbe) as described previously (Hernandez‐Miranda et al., 2011). Briefly, 27 µl serum‐free dissociation media or 1% FBS dissociation media was placed into the lower compartment of the chamber. Dissociated MGE cells were resuspended in serum‐free medium (10^5^ cells/50 µl) and placed in wells of the upper compartment of the chamber. These were separated from the lower chamber by a polycarbonate porous membrane (8‐µm pores), precoated with laminin (10 µg/ml). The chamber was kept in an incubator at 37°C overnight. After incubation, the migrated cells that adhered to the underside of the membrane were fixed and stained using the Diff‐Quick kit (Reagena). For quantitative analysis, the membranes were observed with an Olympus light microscope with a ×20 objective adapted with a 500 × 500‐µm grid. Four random fields of stained cells were counted for each well, and the mean number of migrating cells per square millimeter for each experimental condition was estimated.

### Adhesion assay

MGEs were isolated from E12.5 *PlexinA1^+/^^+^* and *PlexinA1^−/−^* littermates and dissociated cultures prepared as described above. Cell densities were adjusted to 2 × 10^6^/ml in culture medium, and 50 µl cell suspension was seeded onto coated coverslips in triplicate. The coverslips were incubated at 37°C for 30 minutes, washed with PBS, and fixed with 2% PFA for 15 minutes. Attached cells were visualized with DAPI. Six pictures were taken per coverslip (×20 magnification), and attached cells were counted.

### Angle measurements

E14.5 *PlexinA1^+/^^+^* and *PlexinA1^−/−^* coronal brain sections (n = 3 both groups) were stained with the progenitor marker nestin. The degree of variation from the horizontal plane between the tip of apical and basal process within the ventricular zone of the MGE was measured in ImageJ 1.48 software (NIH; RRID: nif‐0000–30467).

### In utero electroporation

In utero electroporation of the MGE was performed as described previously (Wu et al., 2011). Briefly, timed pregnant C57/BL6 mice at E12.5 were anesthetized, the abdomen opened, and the uterus was exposed. DNA vectors (1 µg/µl, 2 µl; siRNA) were injected into the third ventricle of embryos through a glass micropipette and introduced into the ventricular zone of the MGE by delivering electric pulses (40 V, 50 msec, 4 Hz) through the uterus. The uterus was repositioned in the abdominal cavity, and the abdominal wall and skin were sewn with surgical sutures. The embryos were fixed in PFA at E14.5. We quantified the number of GFP‐positive cells (in utero electroporated animals) in the MGE that were attached/unattached to the ventricular zone surface (n = 3/4 embryos for each siRNA construct).

### Semaphorin binding assays

Alkaline phosphatase (AP)‐conjugated Sema3A (SEMA3A‐AP) was prepared as previously described (Vieira et al., 2007). Fresh frozen E14.5 brain sections were fixed in absolute methanol for 5 minutes, washed five times with PBS, incubated in PBS containing 0.1% Triton X‐100 (PBT) and 10% FBS for 30 minutes, and then reacted with SEMA3A‐AP at RT for 2 hours. Sections were then washed for 5 minutes with PBS, fixed in PFA at RT for 2 minutes, and washed again. Endogenous AP was heat inactivated by incubation at 65°C for 3 hours. Tissue‐bound, heat‐stable recombinant AP activity was detected as an insoluble reaction product after incubation with NBT and BCIP.

### Digital image acquisition and processing

Brightfield and fluorescent images were collected with a light‐ or confocal microscope (Leica). Images were reconstructed and digitized in Photoshop CS2 (Adobe Systems, Mountain View, CA; RRID: SciRes_000161).

### Statistical analysis

Statistical analyses were performed in GraphPad 3 (Graph‐Pad Software, San Diego, CA; RRID: nlx_156835). All data are reported as mean ± SEM. The statistical significance between group means was tested by one‐way ANOVA, followed by Bonferroni's post hoc test (for multiple‐comparisons tests). Significance was set at *P* = 0.05.

## RESULTS

### Expression of *PlexinA1* in migrating interneurons

Previous studies have shown a function for Nrp receptors in cortical interneuron development but no clear role for their coreceptor, PlexinA1. To address this point, we first compared the expression of PlexinA1 relative to the interneuron marker Lhx6 (Alifragis et al., 2004) throughout early (E13.5), middle (E15.5), and late (E18.5) stages of corticogenesis.

**Figure 1:**
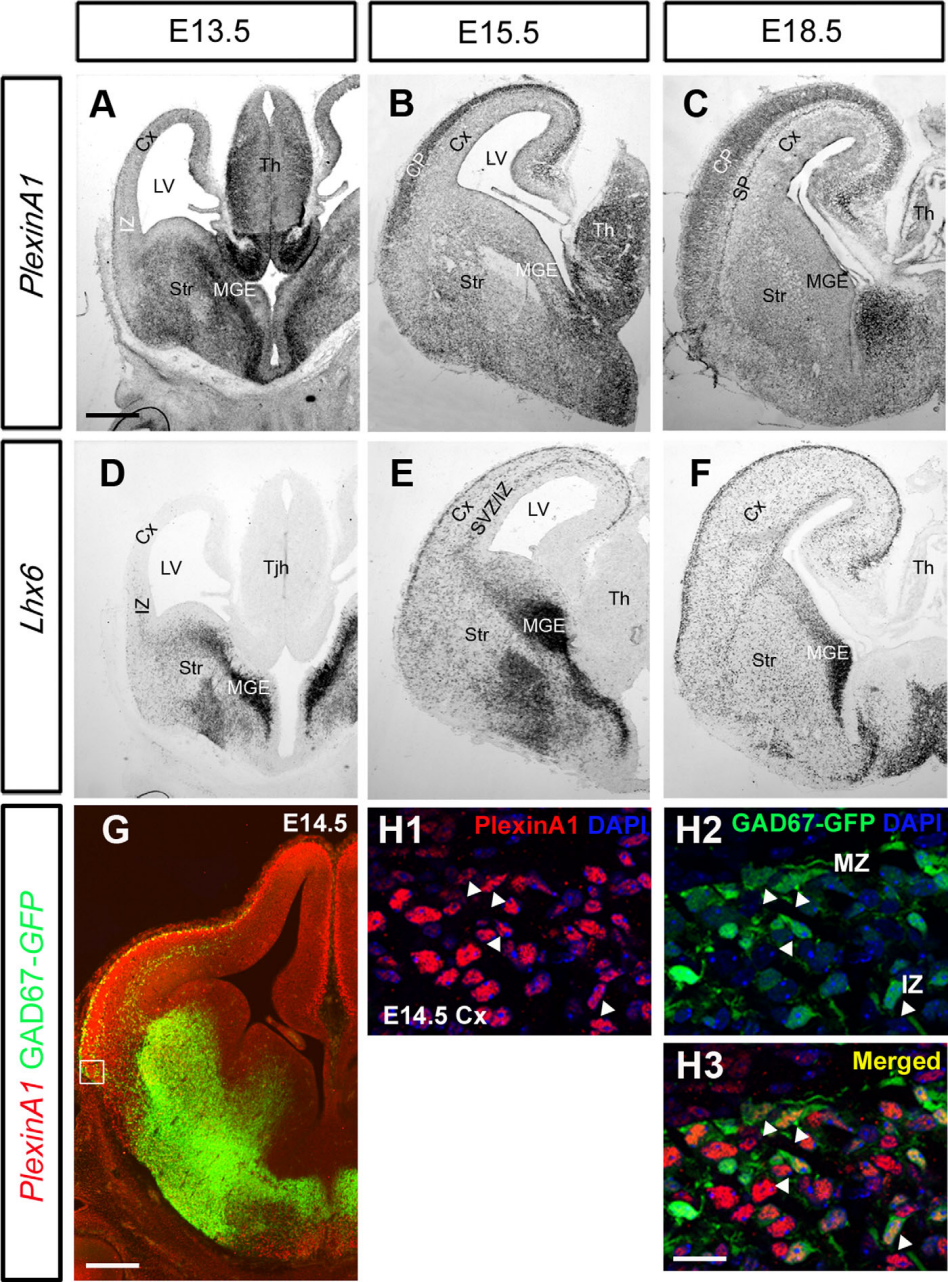
Expression patterns of *PlexinA1* and *Lhx6* in wild‐type mouse brain. **A–F:** In situ hybridization on coronal sections at E13.5 (A,D), E15.5 (B,E), and E18.5 (C,F) for *PlexinA1* (A–C) and the interneuron marker *Lhx6* (D–F). Overlapping patterns of expression were observed between *PlexinA1* and *Lhx6* at all ages. **G–H3:** Coronal section through the brain of a E14.5 GAD67‐GFP mouse, processed for immunohistochemistry with PlexinA1 antibody (red), showed the presence of the receptor within interneurons in the MGE, LGE, MZ, and IZ in the cortex (arrows H1–H3). CP, cortical plate; SP, subplate; Cx, cerebral cortex; IZ, intermediate zone; LV, lateral ventricle; MGE, medial ganglionic eminence; Str, striatum; SVZ, subventricular zone; Th, thalamus. Scale bars = 200 µm in A (applies to A–F); 200 µm in G; 30 µm in H.

At E13.5, the expression pattern of *PlexinA1* overlapped partially with *Lhx6* in the intermediate zone (IZ) of the cortex and more extensively in the mantle zone of the ventral telencephalon (Fig. 1A,D). *PlexinA1* expression was also observed in the ventricular zone (VZ) and subventricular zone (SVZ) of the MGE. At E15.5, it showed a high degree of overlap with *Lhx6* in the ventral telencephalon, although in the cortex it was strongly expressed in the cortical plate (CP) and overlapped with *Lhx6* in the marginal zone (MZ) (Fig. 1B,E). At the end of corticogenesis (E18.5), *PlexinA1* expression, similar to *Lhx6*, was evident in both dorsal and ventral forebrain (Fig. 1C,F), and in the former it was strongly present in the CP and subplate (SP; Fig. 1C).

To confirm that PlexinA1 is expressed in cortical interneurons, we used coronal sections from E14.5 GAD67‐GFP mice (Tamamaki et al., 2003b). Double labeling for PlexinA1 and GFP revealed colocalization in the mantle zones of the MGE and LGE as well as in the IZ and MZ of the cortex (Fig. 1G). In the cortex, approximately 50% (47.33% ± 4.11%) of GFP^+^ cells (presumptive interneurons) appeared to coexpress PlexinA1 (Fig. 1H1–H3). PlexinA1 expression in interneurons is in concordance with previous PCR (Hernandez‐Miranda et al., 2011) and Western blot analyses of MGE cells isolated by FACS (Andrews et al., 2013). Thus, it appears that cortical interneurons express PlexinA1, both at their sites of origin within the ventral forebrain and along their migratory paths to the cortex, suggesting that it may be crucial for their generation, migration, and development.

### Deletion of *PlexinA1* leads to a reduced number of interneurons in the developing cortex

**Figure 2:**
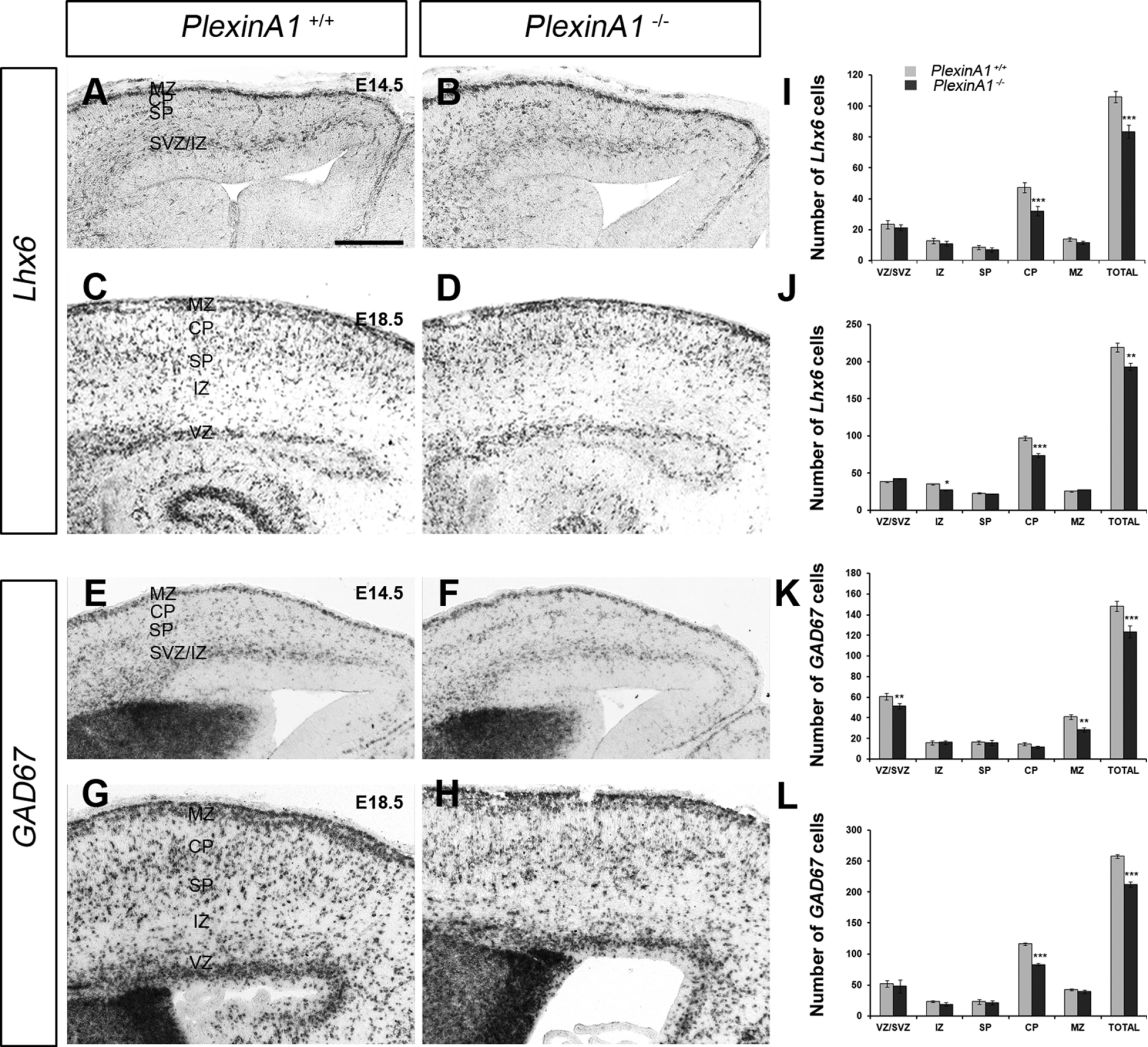
Deletion of PlexinA1 receptor leads to a decreased number of GABAergic interneurons in the cerebral cortex. **A–H:** Photomicrographs of in situ hybridization for *Lhx6* (A–D) and *Gad67* (E–H) on coronal sections through the cortex of *PlexinA1^+/^^+^* (A,C,E,G) and *PlexinA1^−/−^* (B,D,F,H) mice at E14.5 (A,B,E,F) and E18.5 (C,D,G,H). **I–L:** Analysis of the number and distribution of *Lhx6*‐labeled cells at E14.5 (I) and E18.5 (J) and *Gad67* cells at E14.5 (K) and E18.5 (L) in all layers of the cortex of *PlexinA1^+/^^+^* and *PlexinA1^−/−^* mice. Counts were made in the middle region along the rostrocaudal extent of the cortex. Error bars indicate SEM (*P < 0.01, **P < 0.001, ***P < 0.0001). CP, cortical plate; IZ, intermediate zone; MZ, marginal zone; SP, subplate; SVZ, subventricular zone; VZ, ventricular zone. Scale bar = 150 µm.

Using in situ hybridization for the interneuron markers *Lhx6* and *Gad67* (Alifragis et al., 2004; Retaux et al., 1993), we next assessed the number of GABAergic cells within the developing cortex at middle (E14.5) and late (E18.5) developmental stages in *PlexinA1^+/^^+^* and *PlexinA1^−/−^* littermates (n = 6 each). We counted labeled cells in 200‐µm‐wide coronal strips through the middle regions (along the rostral–caudal axis) of the cortex. At E14.5, we observed a significant decrease in the number of both *Lhx6* (*PlexinA1^+/^^+^* 106.12 ± 3.65, *PlexinA1^−/−^*83.47 ± 4.2; *P* < 0.0003) and *Gad67* (*PlexinA1^+/^^+^* 148 ± 4.9, *PlexinA1^−/−^*123.62 ± 5.9; *P* < 0.0002) cells in the cortex of mice lacking the receptor compared with control littermates (Fig. 2A,B,E,F). The reduction in the number of cells positive for both markers was evident across all cortical layers, with the exception of the SP (Fig. 2A,B,E,F).

Analysis at a later stage of corticogenesis (E18.5) similarly revealed significant reductions in the numbers of cells positive for *Lhx6* (*PlexinA1^+/^^+^* 219.34 ± 5.72, *PlexinA1^−/−^* 193.1 ± 4.91; *P* < 0.004) or *Gad67* (*PlexinA1^+/^^+^* 257.8 ± 2.7, *PlexinA1^−/−^* 211.95 ± 4.32; *P* < 0.0006) in the cortex of *PlexinA1^−/−^* mice compared with control littermates across all layers, with the exception of the SP (Fig. 2C,D,G,H). This finding is surprising because PlexinA1 is distinctly expressed in the SP at this age (Fig. 1C). These observations were confirmed by immunohistochemistry for another interneuron marker, calbindin (data not shown). Taken together, these findings suggest alterations in the generation and/or migration of cortical interneurons in *PlexinA1^−/−^* mice.

### No change in migratory potential of *PlexinA1^−/−^* MGE cells

We have recently shown, by using electroporation of siRNAs in brain slices and in utero, that downregulation of either Limk2 or PlexinA1 results in aberrant migration of interneurons through the striatum (Andrews et al., 2013), presumably as a result of perturbed semaphorin signaling. To assess directly whether loss of plexinA1 function affects interneuron migration independently of the responsiveness to semaphorin, we carried out a migratory assay with the Boyden chemotaxis chamber and dissociated MGE cells prepared from E13.5 *PlexinA1^+/^^+^* and *PlexinA1^−/−^* littermates (n = 3 each). In the absence of serum, we observed similar levels of migration between *PlexinA1^+/^^+^* and *PlexinA1^−/−^* mice (*PlexinA1^+/^^+^* 1,264 ± 167 MGE cells/mm^2^, *PlexinA1^−/−^* 1,315 ± 143 cells/mm^2^; *P* < 0.3). Although addition of serum increased the basal level of migration, in line with previous observations (Rakić et al., 2009), we did not observe any significant differences between the two groups (*PlexinA1^+/^^+^* 3,121 ± 447 cells/mm^2^, *PlexinA1^−/−^* 3,251 ± 343 cells/mm^2^; *P* < 0.4), suggesting that loss of PlexinA1 function does not alter the migratory potential of MGE‐derived neurons.

### Reduced number of striatal projection neurons in *PlexinA1^−/−^* mice

**Figure 3:**
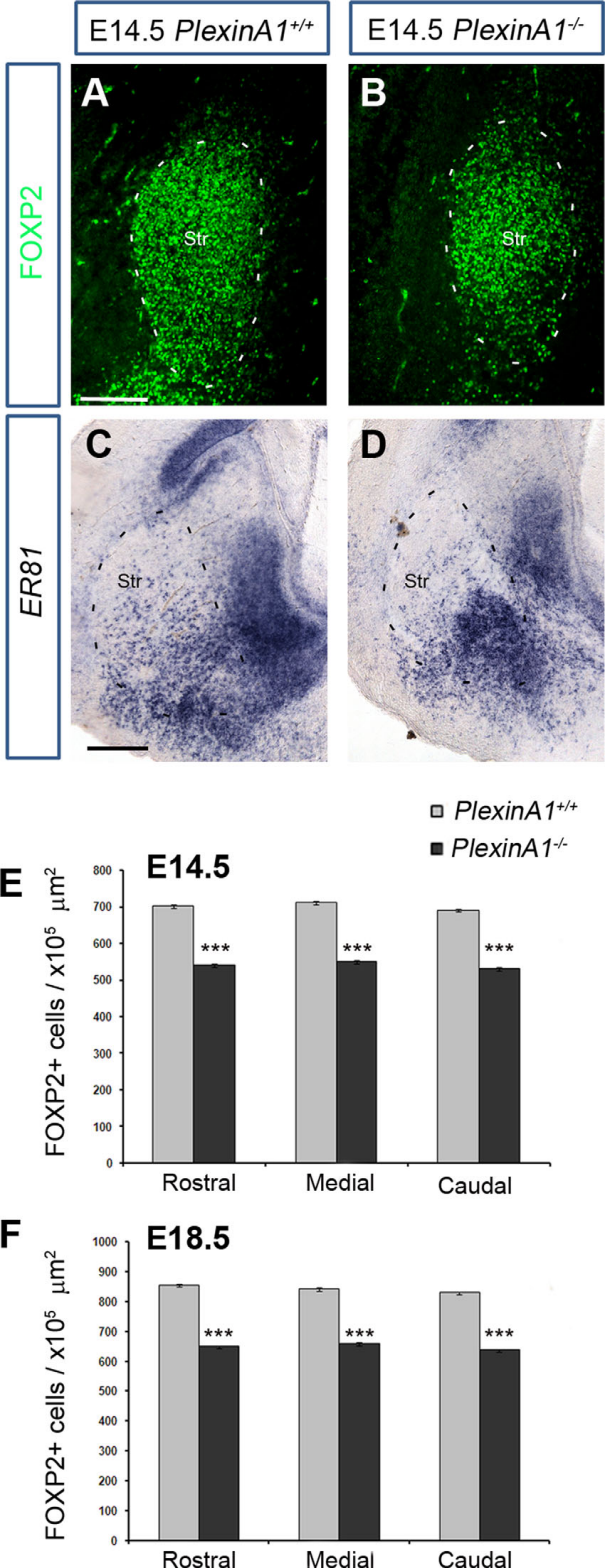
Reduced number of FOXP2^+^ cells in the developing striatum of *PlexinA1^−/−^* mice. Coronal brain sections from *PlexinA1^+/^^+^* (**A,C**) and *PlexinA1^−/−^* (**B,D**) mice at E14.5 were immunostained for FOXP2 (A,B) or processed for in situ hybridization for *ER81* (C,D). Quantification of FOXP2^+^ cells in the striatum of *PlexinA1^−/−^* animals showed reduced numbers of labeled cells compared with *PlexinA1^+/^^+^* littermates at E14.5 (**E**) and E18.5 (**F**). Error bars indicate SEM (***P < 0.0005). Str, striatum. Scale bars = 100 µm in A (applies to A,B); 150 µm in C (applies to C,D).

To assess whether projection neuron numbers were also affected within the striatum, we immunostained coronal sections from *PlexinA1^−/−^* mice and *PlexinA1^+/^^+^* littermates at E14.5 and E18.5 (n = 3 per age for each genotype) for the transcription factor Forkhead box protein P2 (FOXP2), a marker of developing striatal projection neurons (Takahashi et al., 2003). Counts of labeled cells throughout the rostrocaudal extent of the striatum showed a significant reduction in mutants compared with control littermates at E14.5 (medial levels *PlexinA1^+/^^+^* 711.21 ± 4.53 cells/10^5^ µm^2^, *PlexinA1^−/−^* 548.96 ± 4.57 cells/10^5^ µm^2^; *P* < 0.00049) and at E18.5 (*PlexinA1^+/^^+^* 840.45 ± 5.44 cells/10^5^ µm^2^, *PlexinA1^−/−^* 658.75 ± 5.48 cells/10^5^ µm^2^; *P* < 0.00054; Fig. 3A,B,E,F). A similar reduction in the number of striatal projection neurons was observed with another marker, *ER81* (Stenman et al., 2003; Fig. 3C,D).

### Reduced proliferation in the developing forebrain of *PlexinA1^−/−^* mice

**Figure 4:**
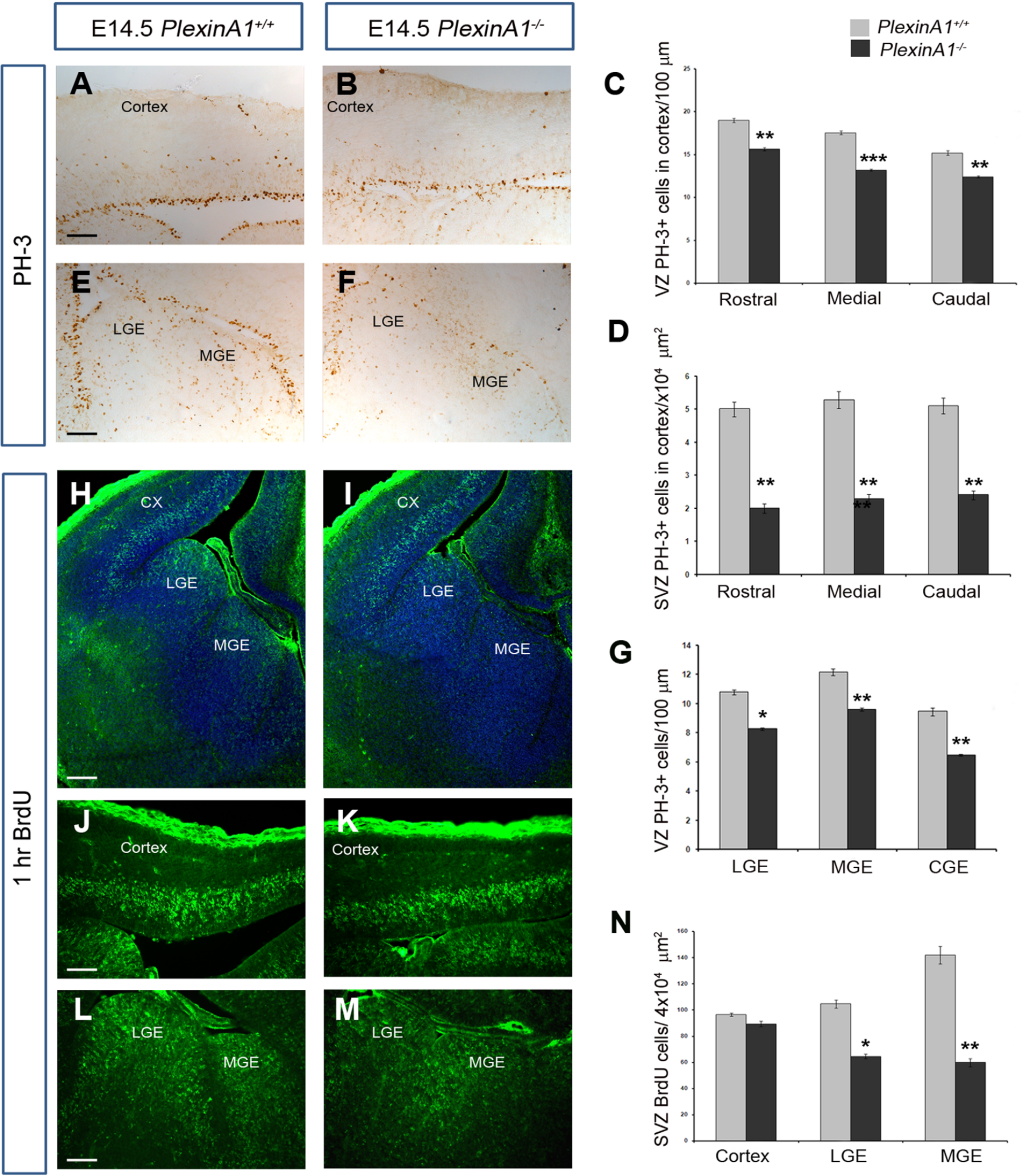
Reduced proliferation in *PlexinA1^−/−^* mice. Coronal brain sections from *PlexinA1^+/^^+^* (**A,E,H,J,L**) and *PlexinA1^−/−^* (**B,F,I,K,M**) mice at E14.5 were immunostained for PH‐3 (A,B,E,F) and BrdU (H–M). Quantification of PH‐3^+^ (**C,D,G**) and BrdU^+^ (**N**) cells in the proliferative zones of *PlexinA1^−/−^* animals showed reduced numbers compared with *PlexinA1^+/^^+^* littermates. Error bars indicate SEM (*P < 0.01, **P < 0.001, ***P < 0.0001). Cx, cortex; CGE, caudal ganglionic eminence; MGE, medial ganglionic eminence; LGE, lateral ganglionic eminence. Scale bars = 50 µm in A (applies to A,B); 150 µm in E (applies to E,F); 150 µm in H (applies to H,I); 50 µm in J (applies to J,K); 150 µm in L (applies to L,M).

The reduced number of cortical interneurons and striatal projection cells could be due to either increased apoptosis or reduced proliferation. To assess these possibilities, we immunostained coronal sections from E14.5 *PlexinA1^−/−^* and control littermate mice (n = 4 for each genotype) for the apoptotic marker cleaved caspase 3 (CC3) and the mitotic marker PH‐3. At this age, we observed very little CC3 staining in the forebrains of control or *PlexinA1^−/−^* mutant mice, in accordance with previous studies (Thomaidou et al., 1997; Yeh et al., 2014), suggesting that the observed reduction in neuronal numbers is unlikely to be due to increased apoptosis. In contrast, analysis of the number of PH‐3‐positive cells in the proliferative zones throughout the rostrocaudal extent of the developing cortex showed a significant decrease in mutants compared with controls (VZ medial level: *PlexinA1^+/^^+^* 17.54 ± 0.20, *PlexinA1^−/−^* 13.19 ± 0.11 per 100 µm [*P* < 0.0002[; SVZ medial level: *PlexinA1^+/^^+^* 5.28 ± 0.26, *PlexinA1^−/−^* 2.28 ± 0.15 per 10^4^ µm^2^ [*P* < 0.0023]; Fig. 4A–D). We also observed a similar decrease in the number of PH‐3 cells along the VZ of the CGE, LGE, and MGE in mutants compared with controls (MGE *PlexinA1^+/^^+^* 12.16 ± 0.24, *PlexinA1^−/−^* 9.59 ± 0.11 100 µm; *P* < 0.0073; Fig. 4E–G).

To confirm these findings, *PlexinA1^−/−^* mice and their wild‐type littermates at E14.5 (n = 4 each) were pulse labeled for 1 hour with BrdU, a thymidine analogue that becomes incorporated into DNA during S phase of the cell cycle. We observed a reduced number of BrdU‐positive cells in *PlexinA1^−/−^* mutant mice compared with control littermates in the LGE (*PlexinA1^+/^^+^* 104.44 ± 3.07, *PlexinA1^−/−^* 64.51 ± 1.87 per 4 × 10^4^ µm^2^; *P* < 0.01) and MGE (*PlexinA1^+/^^+^* 141.76 ± 6.83, *PlexinA1^−/−^* 59.84±.3.12 per 4 × 10^4^ µm^2^; *P* < 0.001; Fig. 4H–N). These observations suggest that the decrease in the numbers of striatal projection neurons and cortical interneurons in the forebrains of PlexinA1‐deficient mice likely is due to reduced proliferation of their progenitors in the LGE and MGE.

**Figure 5:**
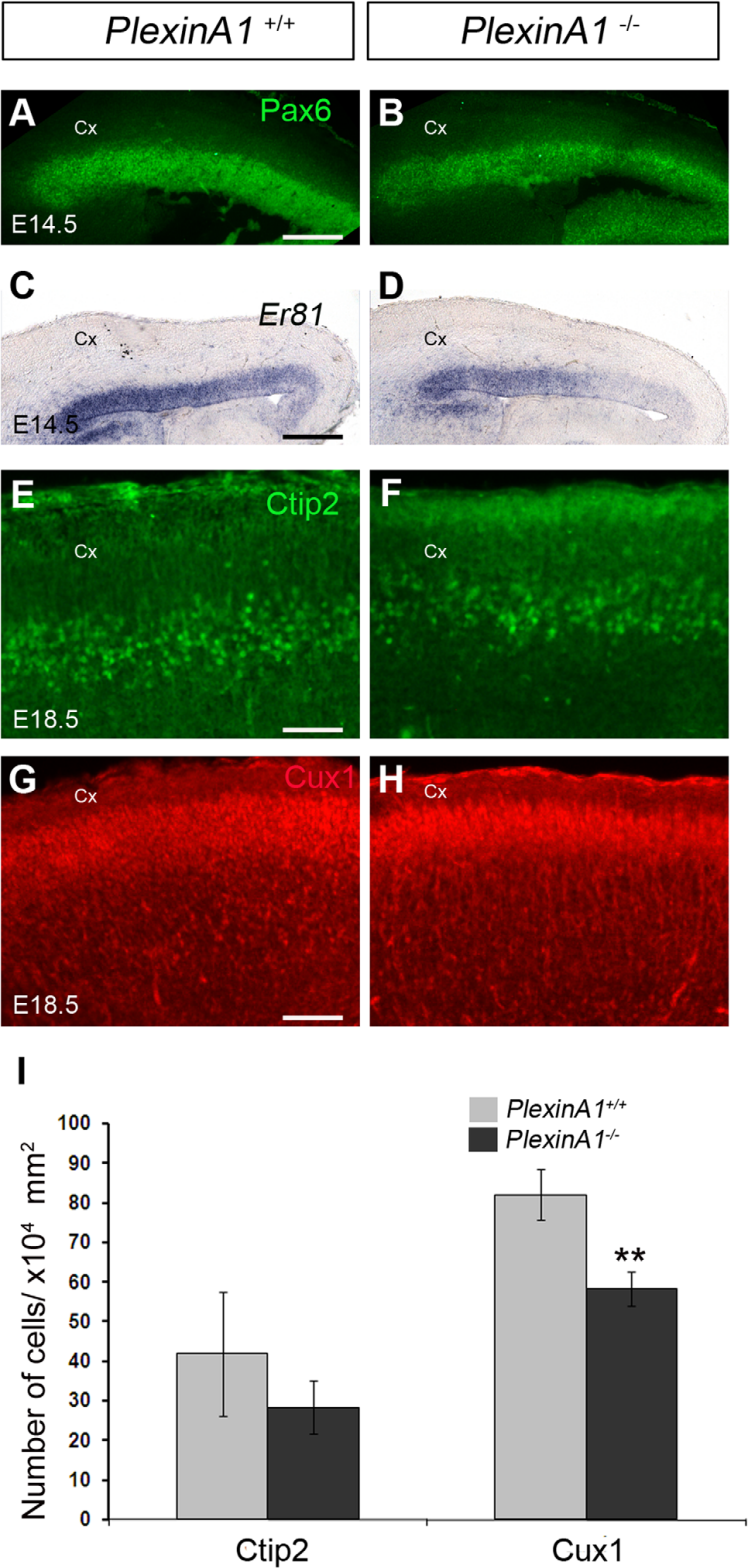
Reduced number of pyramidal neurons in the cortex of *PlexinA1^−/−^* mice. Coronal brain sections from *PlexinA1^+/^^+^* (**A,C,E,G**) and *PlexinA1^−/−^* (**B,D,F,H**) mice at E14.5 (A–D) and E18.5 (E–H) were immunostained for Pax6 (A,B), Ctip2 (E,F), and Cux1 (G,H) or processed for in situ hybridization for *Er81* (C,D). **I:** Quantification of Ctip2 and Cux1^+^ cells in the cortex of *PlexinA1^−/−^* animals showed reduced numbers compared with *PlexinA1^+/^^+^* littermates. Error bars indicate SEM (**P < 0.001). Cx, cortex. Scale bars = 200 µm in A (applies to A,B); 200 µm in C (applies to C,D); 100 µm in E (applies to E,F); 100 µm in G (applies to G,H).

To assess whether the decreased number of mitotically active progenitor cells in the cortex of *PlexinA1^−/−^* mice results in a decrease in pyramidal neurons, we carried out immunostaining for the cortical progenitor cell marker Pax6 and in situ hybridization for its downstream target, *ER81*, a layer 5 neuronal marker (Tuoc and Stoykova, 2008) at E14.5. We observed reduced expression of both markers in the cortex of *PlexinA1^−/−^* mice compared with control littermates (n = 3, both groups; Fig. 5A–D). We also observed a significantly reduced number of Ctip2 (early‐born)‐ and Cux1 (late‐born)‐positive neurons (Arlotta et al., 2005; Cubelos et al., 2008) in the developing cortex at E18.5 (Ctip2: *PlexinA1^+/^^+^* 41.93 ± 15.7, *PlexinA1^−/−^* 28.41 ± 6.7 [*P* < 0.3]; Cux1: *PlexinA1^+/^^+^* 82.07 ± 6.4, *PlexinA1^−/−^* 58.24 ± 4.3 [*P* < 0.005]; Fig. 5E–I). This reduction correlated with significantly decreased cortical thickness (*PlexinA1^+/^^+^* 711.6 ± 6.04, *PlexinA1^−/−^* 666 ± 11.72 µm; *P* < 0.07). Thus our findings suggest that loss of PlexinA1 function impairs the generation of neurons contributing to the cortex.

**Figure 6:**
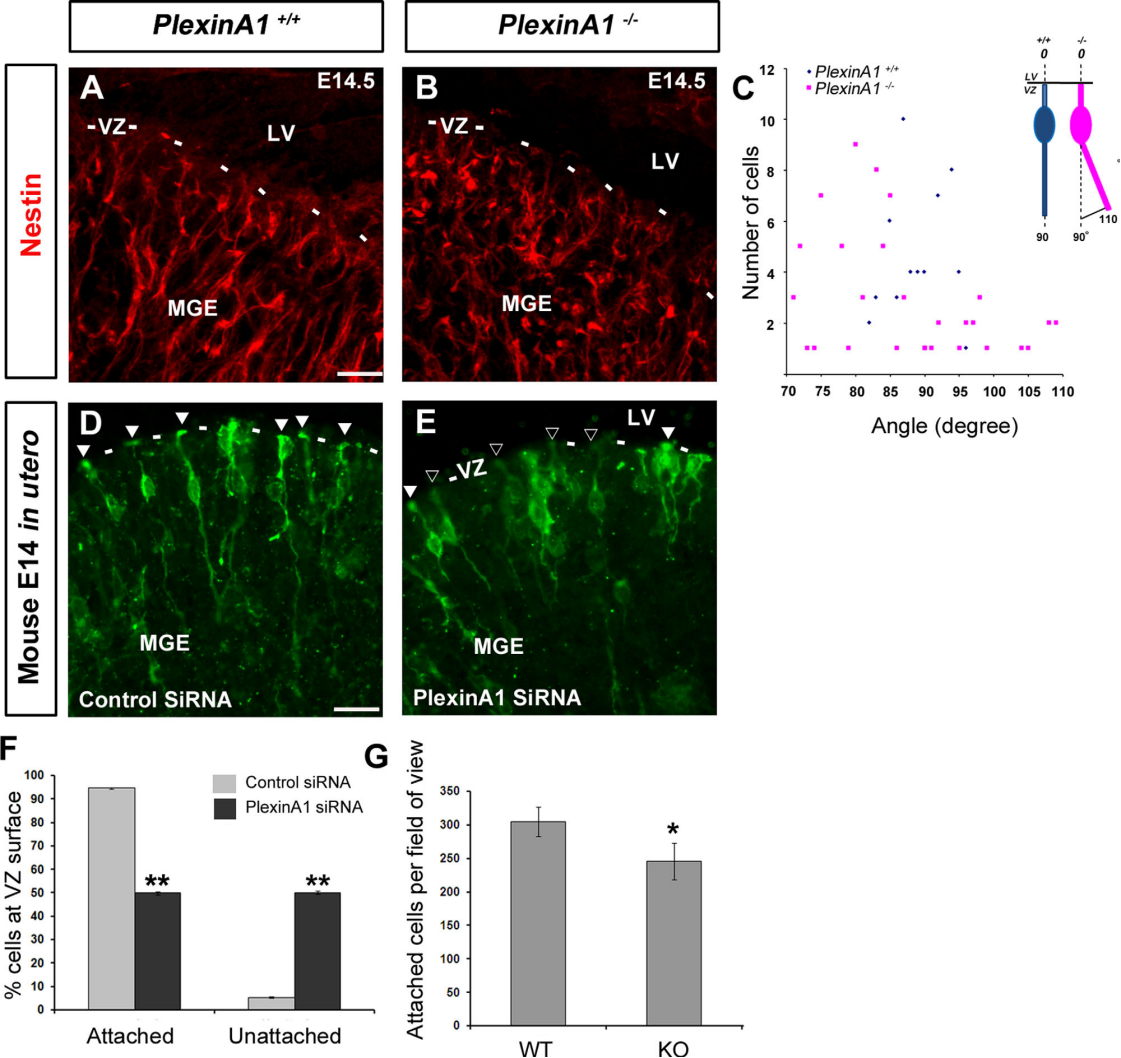
Reduced attachment and altered morphology of progenitor cells in the MGE of *PlexinA1^−/^^−^* mice. Coronal brain sections from *PlexinA1^+/^^+^* (**A**) and *PlexinA1^−/−^* (**B**) mice at E14.5 were immunostained for the progenitor cell marker Nestin (**A‐B**). (**A,B**) Fewer Nestin‐positive cells appear to be attached to the ventricular wall and positioned perpendicular to the ventricular surface in *PlexinA1^−/^^−^* mice. (**C**) Quantification of the orientation of the basal process of labelled cells in the VZ/SVZ in *PlexinA1^−/^^−^* mice. (**D,E**) Mouse embryos electroporated in utero at E12.5 with control‐(GFP)siRNA (**D**) or PlexinA1‐(GFP)siRNA (**E**) into the MGE and harvested at E14.5. Fewer cells appear to be attached to the ventricular surface following PlexinA1 knockdown (black arrowheads) compared to control (white arrowheads), quantified in F. (**G**) Quantification of adhesion assay, showing fewer E12.5 dissociated MGE cells from *PlexinA1^−/^^−^* mice attached to coated coverslips compared to control littermates. Scale bars in A‐B, 20 μm; and D‐E, 50 μm. (*P < 0.01). Error bars indicate SEM. Abbreviation LV, lateral ventricle; MGE, medial ganglionic eminence; VZ, ventricular zone.

To explore further the apparent decline in proliferation in the MGE, we examined the morphology of progenitors cells along the VZ by immunostaining E14.5 ventral forebrain sections of *PlexinA1^+/^^+^* and *PlexinA1^−/−^* mice (n = 3 both groups) with an antibody to the neuronal progenitor marker nestin. As expected, nestin‐positive cells from *PlexinA1^+/^^+^* mice appeared to be anchored and positioned perpendicular to the ventricular surface (52/57 cells, corresponding to 91.23%, were located at 85–95°); in contrast, in *PlexinA1^−/−^* mice, fewer nestin‐positive cells were attached to the ventricular wall, and fewer were positioned perpendicular to the ventricular surface (17/79 cells, corresponding to 21.52%, were located at 85–95°; n = 3 each; Fig. 6A–C). There were also fewer nestin‐positive cells in *PlexinA1^−/−^* mice, in agreement with our PH‐3 data. The lack of receptor appeared to have a similar effect on the number and orientation of progenitor cells in the VZ of the dorsal forebrain (data not shown).

To determine whether the observed effect is cell autonomous, we carried out in utero electroporation of PlexinA1 and control GFP‐siRNA constructs into the MGE (n = 4 both groups). We quantified the percentage of GFP‐positive cells (186 control siRNA and 260 PlexinA1 siRNA cells) that were anchored to the ventricular surface. Most control GFP‐labeled cells (94.5% ± 0.27%) appeared to have pronounced end feet, anchored to the ventricular wall (white arrowheads, Fig. 6D), and long radial processes; in contrast, PlexinA1‐knockdown cells were infrequently attached to the ventricular surface (49.87% ± 0.68%, *P* < 0.003) and had shorter and thinner processes (black arrowheads, Fig. 6E). This finding is consistent with a previous study that demonstrated reduced proliferation and alteration of progenitor cell morphology in response to inflammation (Stolp et al., 2011).

Plexins were initially identified as cell adhesion molecules (Ohta et al., 1995), so we wondered whether loss of PlexinA1 function reduced attachment of neural progenitors to the extracellular matrix at the ventricular surface. We observed that fewer nestin‐positive cells from *PlexinA1^−/−^* mice attached to laminin/poly‐L‐lysine‐coated coverslips 30 minutes after seeding, compared with cells from control littermates (n = 3 both groups; Fig. 6G; *PlexinA1^+/^^+^* 305.17 ± 21.78, *PlexinA1^−/−^* 245.78 ± 27.07; *P* < 0.007). These observations suggest that PlexinA1 promotes cell adhesion to maintain the number and correct arrangement of progenitor cells in the developing forebrain.

## DISCUSSION

Cortical interneurons, generated predominantly in the MGE, migrate through the ventral and dorsal telencephalon before reaching their final positions within the CP (Métin et al., 2006; Faux et al., 2012). Previously we demonstrated that interneurons in Robo1 knockout (*Robo1^−/−^*) mice express reduced levels of Nrp1 and PlexinA1, rendering them less responsive to the chemorepulsive effects of Sema3A and Sema3F and causing their aberrant migration through the developing striatum en route to the cortex (Hernandez‐Miranda et al., 2011). Even though earlier studies had demonstrated the importance of Nrps in this process (Marin et al., 2001; Tamamaki et al., 2003a), a potential role for PlexinA1 in interneuron development had not previously been shown.

To complement and extend a previous study (Perälä et al., 2005) demonstrating PlexinA1 mRNA expression in the developing mouse forebrain, we used in situ hybridization to define its precise expression pattern. These experiments revealed the presence of PlexinA1 in progenitor cells lining the ventricular surface of the ganglionic eminences as early as E13.5 and in migrating interneurons expressing GAD67. Consistent with an important role for PlexinA1 in interneuron development, a significant decrease in the number of these cells in the cortex of *PlexinA1^−/−^* mice at E14.5 and E18.5 was observed. We first considered the possibility that this might be due to altered migration, but our migration assay failed to detect any significant differences in their migratory potential. We also considered whether changes in apoptosis or proliferation contribute to the decreased number of cortical interneurons in *PlexinA1^−/−^* mice. Previous studies have highlighted the importance of programmed cell death in shaping the cortex throughout development (Thomaidou et al., 1997; Haydar et al., 1999). However, we observed little cell death in the cortex of *PlexinA1^−/−^* mice, suggesting that increased apoptosis is unlikely the cause for the reduced interneuron number. We then considered the possibility of alterations in proliferation of forebrain progenitors of mice lacking the receptor. Plexins have been shown to play key roles in regulating cytoskeletal architecture and cell proliferation. PlexinA1 or PlexinA4 knockdown studies in endothelial cells resulted in prominent rearrangements of the actin cytoskeleton that were accompanied by inhibition of cell proliferation (Kigel et al., 2011). More recently, PlexinB2 has been shown to regulate the proliferation and migration of neuroblasts in the postnatal and adult SVZ (Saha et al., 2012). Consistent with such role for PlexinA1 in forebrain progenitor cells, reduced proliferation and progenitor cell numbers in the cortex, LGE, and MGE of *PlexinA1^−/−^* mice was observed, which would account for the reduction in the number of cortical pyramidal neurons and interneurons and of striatal projection cells in these mice.

Plexins and their coreceptors, Nrps, play important roles in vascular as well as neuronal development (Vieira et al., 2007; Gu and Giraudo, 2013). Thus, we wondered whether altered blood vessel formation within the developing forebrain of PlexinA1 mutant mice might contribute to the observed decrease in neurogenesis. However, staining for vascular markers did not show any obvious changes in the number or patterning of blood vessels in the developing forebrain in mutants compared with control littermates (data not shown). This observation indicated that vascular patterning is relatively normal in these mice and is unlikely to contribute to altered neurogenesis. However, our findings do not exclude the possibility of a more intimate interaction between vasculature and progenitors. This can be studied only with detailed analysis of the vasculature together with the progenitors, similar to that described in a study by Stubbs et al. (2009), who charted the movements and neurite extensions of live, individually labeled neuronal progenitors and/or newly born neurons in cortical slice cultures in relation to labeled vasculature.

**Figure 7:**
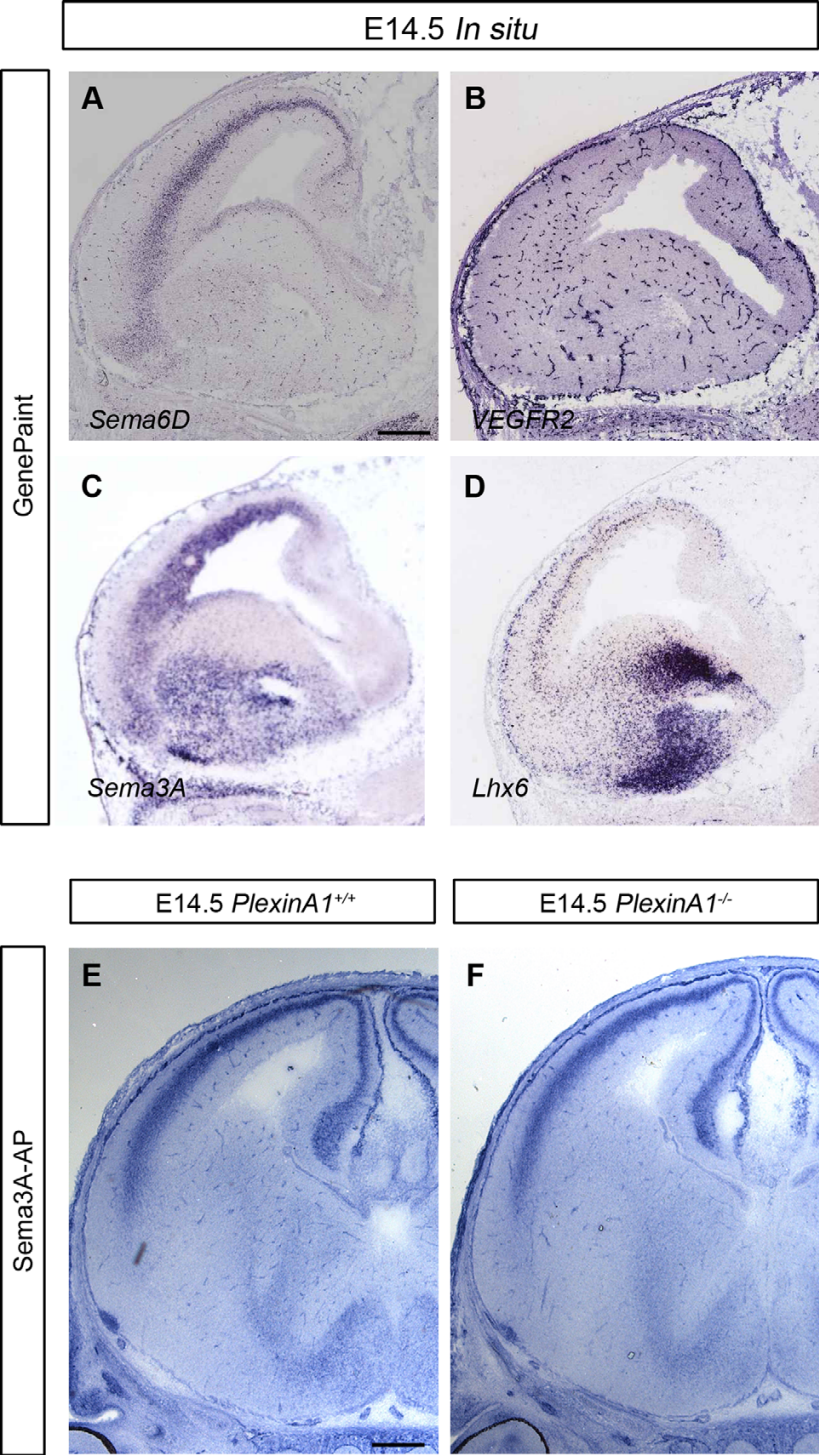
Expression patterns of semaphorins and VEGFR2 in wild‐type mouse brain. **A–D:** In situ hybridization of parasagittal sections at E14.5 for *Sema6D* (A) *VEGFR2* (B), *Sema3A* (C), and the interneuron marker *Lhx6* (D). Images were downloaded from the GenePaint server. **E,F:** Sema3A‐AP was added to coronal brain sections from *PlexinA1^+/^^+^* (E) and *PlexinA1^−/−^* (*F*) mice at E14.5. Similar levels of Sema3A‐AP binding were observed in both sets of animals. Scale bars = 300 µm in A (applies to A–D); 200 µm in E (applies to E,F).

PlexinA1 has been shown to have a function in different semaphorin signaling pathways. Thus, it acts as receptor for Sema6D and forms a complex with the vascular endothelial growth factor receptor 2 (VEGFR2) to convey signals for this semaphorin during cardiac morphogenesis (Toyofuku et al., 2004). Furthermore, a complex formed by Sema3A, Nrp1, and PlexinA1 signaling promotes lymphatic valve formation (Bouvrée et al., 2012). In the nervous system, perturbations in this signaling pathway result in defects in the projection of statoacoustic ganglion neurons (Katayama et al., 2013). We, therefore, examined the expression of semaphorins and VEGF receptors within the developing forebrain using data available in the GenePaint database. Sema6D and VEGFR2 both appeared to be expressed in the vasculature, and not in the proliferative zones (Fig. 7A,B), suggesting that they are unlikely to play a role in the present PlexinA1‐mediated events. Also, Sema3A was expressed very strongly throughout the cortex, in the VZ of the LGE, and along the routes of migrating interneurons (Fig. 7C,D). In contrast, it was only weakly expressed in the VZ of the MGE. To examine whether a defective Sema3A signaling pathway might be involved in proliferation, we carried out a Sema3A‐AP binding assay, which failed to show any differences between wild‐type and *PlexinA1^−/−^* mice (Fig. 7E,F). Thus, although we cannot definitively rule out involvement of these two semaphorin signaling pathways, this finding indicates that the effect on proliferation from loss of PlexinA1 function is likely to occur via another mechanism(s).

Slit–Robo and semaphorin–plexin signaling pathways participate in various developmental and pathogenic processes. We have previously described a cross‐talk between the two signaling pathways, with Robo1 playing a role in semaphorin signaling within the ventral forebrain, in part by regulating the expression of Nrp and plexin receptors in interneurons in *Robo1^−/−^* mice (Hernandez‐Miranda et al., 2011). PlexinA1 has also recently been shown to modulate Slit signaling (Delloye‐Bourgeois et al., 2015). We and others have shown that Slit–Robo signaling plays a role in neurogenic events within the developing forebrain (Borrell et al., 2012; Yeh et al., 2014). These findings raise the possibility that PlexinA1 might also modulate Slit signaling during corticogenesis. However, we think this is unlikely because Slit and PlexinA1 mutants affect neurogenesis differently, with an increased number of proliferating progenitors in the forebrain of mice lacking both Slit1 and Slit2 or Robo1 (Yeh et al., 2014) and fewer in *PlexinA1^−/−^* mice.

Complex interactions between neural stem/progenitor cells and extracellular matrix (ECM) proteins regulate their proliferation and differentiation, and many of these interactions involve transmembrane integrin receptors (Wojcik‐Stanaszek et al., 2011). The PlexinA1 coreceptor Nrp1 was also originally identified as a surface protein mediating cellular adhesion (Shimizu et al., 2000). Nrp1 was found to interact with β1 integrin in pancreatic carcinoma cells and to be important for integrin‐mediated anchorage‐independent growth and adhesion (Fukasawa et al., 2007). Knockdown of Nrp1 has also been shown to inhibit endothelial cell adhesion to fibronectin by altering the functional activity of α5β1 integrin (Valdembri et al., 2009). Plexins were also initially identified as cell surface molecules, and their exogenous expression on the surface of L cells promotes cell adhesion via a homophilic binding mechanism (Ohta et al., 1995; Fujisawa et al., 1997). Subsequent studies have shown that Sema4D activation of PlexinB1 and PlexinA1 hinders cell attachment to adhesive substrates, resulting in cell collapse and inhibition of cell migration (Barberis et al., 2004).

Dissociated neuronal progenitor cells attach to tissue culture dishes or glass coverslips only when coated with a permissive substrate such as laminin. Thus, reduced adhesion of PlexinA1 knockdown cells would suggest that loss of receptor function perturbs attachment to laminin. Plexins associate with integrins (Basile et al., 2007; Choi et al., 2014), which are known laminin receptors (Tomaselli et al., 1988). Thus, it is possible that PlexinA1 could be acting as an integrin coreceptor required for laminin binding and attachment to the ECM. Interestingly, ephrin B1 signaling has been shown to enhance ECM adhesion of neuroepithelial cells by promoting apical localization of integrin β1 (Arvanitis et al., 2013). By analogy, loss of PlexinA1 function might impair the apical distribution of integrins in progenitor cells and, thereby, affect their ability to attach properly to the ECM and consequently impair their capacity to divide. Further studies will be required to assess the possibility that PlexinA1 regulates integrin function in cortical neural progenitors.

In summary, we report here that progenitor cells lining the telencephalic ventricles express PlexinA1 and that absence of this receptor impairs neurogenesis in the developing forebrain, likely because of impaired ECM attachment of neural progenitors to the ventricular surface. Ultimately, this defect hinders the formation of striatal projection neurons and cortical pyramidal cells and interneurons during corticogenesis.

## CONFLICT OF INTEREST STATEMENT

The authors declare no conflicts of interest.

## ROLE OF AUTHORS

Designed the research: WDA, JGP. Performed the research: WDA, KD, NT. Analyzed data: WDA. Wrote the manuscript: WDA, CR, JGP
